# Evaluation of ctDNA-guided adjuvant therapy de-escalation in head and neck squamous cell carcinoma: a comparative cohort study

**DOI:** 10.3389/fimmu.2025.1576042

**Published:** 2025-08-07

**Authors:** Xu Zhang, Qigen Fang, Junhui Yuan, Liyuan Dai, Ruihua Luo, Tao Huang, Yinfei Wu

**Affiliations:** 1 Department of Head Neck and Thyroid, The Affiliated Cancer Hospital of Zhengzhou University & Henan Cancer Hospital, Zhengzhou, China; 2 Department of Radiology, The Affiliated Cancer Hospital of Zhengzhou University & Henan Cancer Hospital, Zhengzhou, China; 3 Department of Breast, The Affiliated Cancer Hospital of Zhengzhou University & Henan Cancer Hospital, Zhengzhou, China; 4 Moores Cancer Center, University of California, San Diego, San Diego, CA, United States; 5 Health Management Center, The Affiliated Cancer Hospital of Zhengzhou University & Henan Cancer Hospital, Zhengzhou, China

**Keywords:** head and neck squamous cell carcinoma, CtDNA, neoadjuvant chemoimmunotherapy, adjuvant chemotherapy, pathologic complete response

## Abstract

**Background:**

While circulating tumor DNA (ctDNA) assessment after surgery has emerged as a promising biomarker for minimal residual disease detection in solid tumors, its clinical utility for guiding the selection between postoperative radiotherapy (PORT) and chemoradiotherapy (POCRT) in head and neck squamous cell carcinoma (HNSCC) remains poorly characterized. We evaluated whether ctDNA-directed stratification could optimize locoregional control in HNSCC patients following neoadjuvant chemoimmunotherapy.

**Methods:**

In this comparative cohort study, consecutive HNSCC patients treated with neoadjuvant chemoimmunotherapy were stratified into two management groups: a ctDNA-guided cohort where tumor-informed ctDNA testing determined POCRT administration given only for detectable ctDNA, and a traditional cohort where all patients received PORT, with postoperative chemotherapy decisions made by multidisciplinary team review based on pathologic response status and pretreatment imaging findings. The primary endpoint was 3-year locoregional control, with secondary analysis of POCRT utilization rates.

**Results:**

Among 257 patients who completed neoadjuvant chemoimmunotherapy, 209 (81.3%) underwent surgery with 187 (72.8% of treated patients) achieving major pathological response and comprising our study population. Of these, 69 (36.9%) received ctDNA-guided management, while 118 (63.1%) followed traditional protocols. POCRT utilization was significantly lower in the ctDNA-guided group (27.5% [19/69] vs 42.4% [50/118]; absolute difference -14.9%, p=0.042). ctDNA positivity rates were comparable between groups (ctDNA-guided: 27.5% [19/69] vs traditional: 29.6% [35/118], p=0.867). ctDNA-guided management demonstrated superior outcomes, with a 15% reduction in locoregional recurrence risk (adjusted HR 0.85, 95%CI 0.70-0.94; p=0.013) versus traditional management. Among ctDNA-positive patients, POCRT benefit was significantly greater in the ctDNA-guided cohort (HR 0.73, 95%CI 0.57-0.83; p=0.026) compared to ctDNA-positive patients receiving traditional management (HR 0.87, 95%CI 0.73-0.93; p=0.047; interaction p=0.039).

**Conclusion:**

Postoperative ctDNA analysis identifies HNSCC patients who benefit most from POCRT, enabling a 41% relative reduction in treatment utilization while maintaining superior locoregional control. The enhanced therapeutic effect observed in ctDNA-guided patients supports ctDNA’s role as a decision-modifying biomarker for personalization management following neoadjuvant chemoimmunotherapy.

## Introduction

Head and neck squamous cell carcinoma (HNSCC) ranks as the sixth most prevalent malignancy worldwide, with >50% of cases presenting as locally advanced disease at diagnosis (1). Current standard-of-care for resectable advanced HNSCC involves multimodal therapy combining surgical resection with risk-adapted postoperative radiotherapy (PORT), where chemoradiotherapy (POCRT) is reserved for patients exhibiting high-risk pathological features including extranodal extension (ENE) or positive margins (2). However, meta-analyses demonstrate only marginal survival benefits from POCRT (absolute improvement 5-8%) despite significantly increased toxicity (3–5), highlighting an unmet need for more precise biomarkers to guide adjuvant therapy selection (6).

The therapeutic paradigm for HNSCC has undergone transformative change with immune checkpoint inhibitors. While pembrolizumab is now first-line for recurrent/metastatic disease (7), recent phase II trials reveal neoadjuvant chemoimmunotherapy achieves pathological complete response (pCR) rates of 50-60% without delaying surgery (8, 9). This therapeutic advance creates a critical knowledge gap: conventional pathological risk stratification systems may have reduced predictive value following immunotherapy-induced tumor microenvironment modulation.

Circulating tumor DNA (ctDNA) has established clinical validity for minimal residual disease (MRD) detection across solid tumors. Prospective multicenter studies demonstrate that postoperative ctDNA detection predicts recurrence with >90% specificity (positive predictive value 82-94%) in colorectal and breast cancers (10–12). More importantly, randomized trials (DYNAMIC, CIRCULATE) prove ctDNA-guided adjuvant therapy reduces chemotherapy use by 40-50% while maintaining disease-free survival (13–15). However, its utility in HNSCC, particularly after neoadjuvant chemoimmunotherapy, remains unexplored.

Based on these evidence gaps, we hypothesized that quantitative postoperative ctDNA analysis would outperform conventional pathological criteria in identifying HNSCC patients deriving maximal benefit from POCRT following neoadjuvant chemoimmunotherapy, and enabling safe de-escalation of adjuvant therapy in ctDNA-negative patients. Therefore, our goal was to evaluate whether ctDNA-directed POCRT improves locoregional control (LRC) compared to traditional, pathology-driven management.

## Patients and methods

### Ethical approval

This study was approved by Henan Cancer Hospital Institutional Research Committee, and written informed consent for medical research was obtained from all patients before starting the treatment. All procedures were performed in accordance with the relevant guidelines and regulations.

### Research design

**Figure 1:**
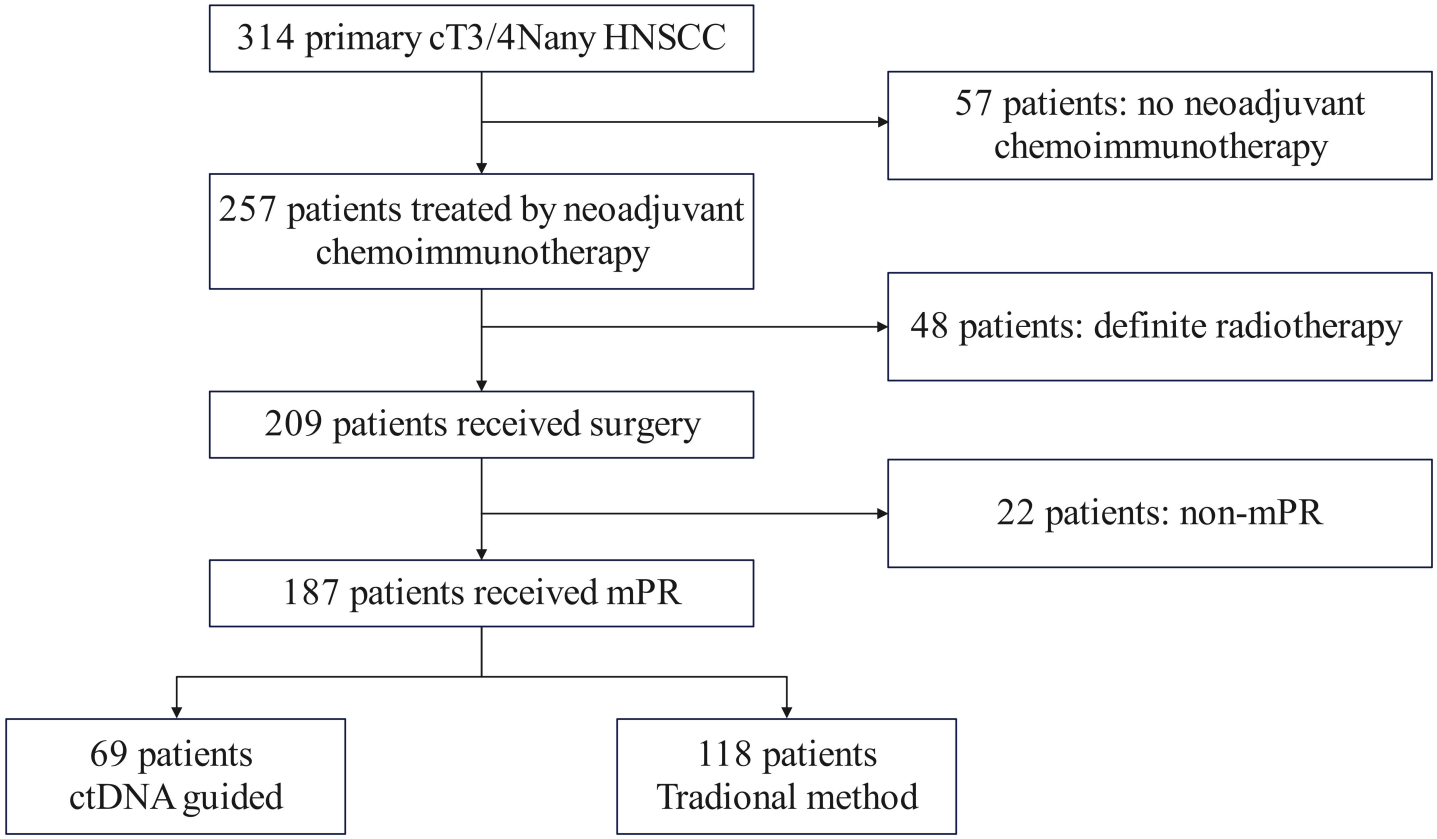
Patient enrollment flow chart.

We performed a retrospective analysis of prospectively collected data from consecutive cT3/4N_any_M0 HNSCC patients treated at a tertiary cancer center between January 2020 and December 2022. Eligible patients met the following criteria: histologically confirmed primary HNSCC, planned for curative-intent neoadjuvant therapy, ECOG 0-1, adequate organ function, and a major pathologic response (mPR) obtained, while excluding those with prior radiotherapy or synchronous malignancies. Following neoadjuvant chemoimmunotherapy, 257 patients proceeded to surgery, with 187 achieving mPR. Postoperative treatment allocation followed a non-randomized, preference-based design. Through structured shared decision-making involving patients, their families, and multidisciplinary tumor boards, 69 patients (36.9%) opted for ctDNA-guided management with POCRT administered only for ctDNA-positive cases, while 118 patients (63.1%) selected standard pathology-driven management (**Figure 1**).

### Variable definition

Tumor staging followed the AJCC Cancer Staging Manual (8^th^ edition). Pathologic response was assessed by dedicated head and neck pathologists using hematoxylin and eosin-stained sections, with mPR defined as <10% viable tumor cells and pCR as no residual invasive or *in situ* tumor in primary and nodal specimens. PD-L1 expression was evaluated using the 22C3 pharmDx assay, with results reported as combined positive score (CPS).

Tumor response to neoadjuvant chemoimmunotherapy was evaluated using contrast-enhanced CT/MRI scans performed at baseline prior to treatment initiation, before each subsequent treatment cycle (every 3 weeks), and at treatment completion according to iRECIST version 1.1 guidelines (16). Response categories were defined as: complete response (CR), partial response (PR), stable disease (SD), and progressive disease (PD). The best overall response for each patient was determined by comparing all serial scans throughout the treatment period.

Treatment-related toxicity was evaluated during both the neoadjuvant and adjuvant therapy phases using the National Cancer Institute Common Terminology Criteria for Adverse Events version 4.0 (17). For both phases, adverse events (AEs) were monitored and recorded: before each treatment cycle through systematic patient interviews and physical examinations; via weekly complete blood counts and biochemistry panels; and at the completion of neoadjuvant therapy through comprehensive laboratory testing and imaging assessments. Toxicity data were collected by treating physicians. The maximum grade observed for each AE during both phases was used for analysis.

Primary outcome variable was the three-year LRC, calculated from the date of surgery to the occurrence of the first locoregional recurrence or the last follow-up.

### ctDNA testing

This methodology aligns with previously published protocols (18). ctDNA was detected in plasma samples gathered prior to neoadjuvant chemoimmunotherapy and within 10-14 days post-surgery. Personalized, tumor-informed ctDNA testing was performed using Illumina (NextSeq 500/NovaSeq 6000) next-generation sequencing platform (Wanlei, Shenyang). Testing was conducted by our core facility, utilizing matched tumor tissue and blood samples to design patientspecific assays. Somatic mutations were identified via multiplex PCR amplification via a 16-plex PCR panel targeting tumor-derived variants, enabling bespoke detection with a sensitivity of 0.01% variant allele frequency. The bioinformatics pipeline included alignment (BWA-MEM), duplex consensus sequencing to reduce noise, and variant calling via a proprietary algorithm, with germline filtering usingmatchedwhite blood cell-derivedDNA. A plasma sample was classified as ctDNA-positive if ≥1 somatic variant was detected. Variant persistence in postoperative samples was confirmed if: the variant was detected pre-treatment, present in the binary comparison map file, or observed in ≥3 reads in postoperative plasma. ctDNA level was recorded with units of mean tumor molecules (MTM) per mL of plasma.

### Treatment

Neoadjuvant regimen comprised the administration of cisplatin at a dosage of 75 mg/m², docetaxel at a dosage of 75 mg/m², and Pembrolizumab or other PD-1 inhibitors including Penpulimab and Tislelizumab at a dosage of 200 mg, spanning two cycles. Surgical intervention was scheduled to occur within one to four weeks following the completion of the six-week neoadjuvant treatment program. The surgical strategy and resection margins were predetermined based on baseline evaluations conducted prior to neoadjuvant therapy and remained unchanged despite treatment responses.

In the ctDNA-guided group, patients received POCRT if ctDNA was detectable and PORT if ctDNA was undetectable. In the traditional treatment group, all patients received PORT, with postoperative chemotherapy decisions made by multidisciplinary team (MDT) review based on pCR status and pretreatment imaging findings.

PORT commenced within six weeks following surgery, precisely targeting the tumor bed with a margin of 1-2 cm and delivering a prescribed dosage of 60-66 Gy. The postoperative chemotherapy employed a platinum-based regimen consisting of 4-6 cycles. Following treatment completion, patients will undergo close monitoring with clinical and radiologic evaluations every 3 months during the first year, then every 3-6 months in the second year. Subsequent follow-ups will occur every 6 months through year 5 to ensure timely detection of potential recurrence.

### Sample size

The sample size was calculated to detect a significant difference in 3-year LRC rates between the ctDNA-guided and traditional treatment groups. Based on preliminary data, we anticipated LRC rates of 85% in the ctDNA group versus 60% in the control group, reflecting a clinically meaningful 25% absolute improvement. Using a two-sided chi-square test with 80% power and a 5% type I error rate (α=0.05), we estimated that 47 patients per group (94 total) would be required to demonstrate this difference. Actual enrollment surpassed this target (69 ctDNA-guided, 118 traditional), providing 96% power for the original effect size and enabling detection of smaller clinically meaningful differences.

### Statistical analysis

Data was descriptively summarized. The chi-square test was used to assess the difference of clinicopathologic variables between ctDNA+ and ctDNA- groups. Survival outcome was compared using univariate and multivariable analysis. All statistical tests were two-sided, with a p-value of less than 0.05 deemed significant. All statistical analyses were conducted using R version 3.4.4.

## Results

### Baseline data

A total of 187 patients were enrolled in the study, with a mean age of 50 ± 15 years. The cohort comprised 140 males (74.9%) and 47 females (25.1%). A history of smoking was reported in 134 patients (71.7%). The primary tumor sites included the oral cavity in 83 patients (44.4%), the oropharynx in 44 patients (23.5%), the larynx in 21 patients (11.2%), and the hypopharynx in 39 patients (20.9%). The clinical tumor stages identified were T3 in 134 patients (71.7%) and T4 in 53 patients (28.3%). Clinical lymph node metastasis was observed in 86 patients (46.0%). Furthermore, 13.4% of the patients exhibited positive expression of p16. Among the cohort, PD-L1 expression was greater than 20 in 68 patients (36.4%) and less than 1 in 23 patients (12.3%).

### Outcome of neoadjuvant therapy

Radiologic evaluations showed that 81 patients (43.3%) achieved a CR, while 106 patients (56.7%) had a PR. Postoperative assessments revealed that 27 patients (14.4%) were classified as ypT1 and 47 patients (25.1%) as ypN+. Negative margins were achieved in all cases, and no pathologic ENE was observed. Prior to neoadjuvant therapy, ctDNA was detectable in all patients, with a median level of 3.00 MTM/ml (range: 0.05-3562.77). After surgery, only 54 patients had detectable ctDNA, with a median level of 1.23 MTM/ml (range: 0.01-67.88), their clinicopathological characteristics showed no significant differences compared to patients without detectable ctDNA (all p>0.05, **Table 1**).

**Table 1:** Baseline data of the 187 patients.

Variable	Overall (N=187)	ctDNA+ (N=54)	ctDNA- (N=133)	P^&^
Age				
<50	74 (39.6%)	22 (40.7%)	52 (39.1%)	
>=50	113 (60.4%)	32 (59.3%)	81 (60.9%)	0.841
Sex				
Male	140 (74.9%)	40 (74.1%)	100 (75.2%)	
Female	47 (25.1%)	14 (25.9%)	33 (24.8%)	0.752
Smoking				
Yes	134 (71.7%)	36 (66.7%)	98 (73.7%)	
No	53 (28.3%)	18 (33.3%)	35 (26.3%)	0.301
Primary site				
Oral cavity	83 (44.4%)	24 (44.4%)	59 (44.4%)	
Oropharynx	44 (23.5%)	13 (24.1%)	31 (23.3%)	
Larynx	21 (11.2%)	6 (11.1%)	15 (11.3%)	
Hypopharynx	39 (20.9%)	11 (20.4%)	28 (21.1%)	0.985
cT				
T3	134 (71.7%)	37 (68.5%)	97 (72.9%)	
T4	53 (28.3%)	17 (31.5%)	36 (27.1%)	0.456
cN				
N0	101 (54.0%)	28 (51.9%)	73 (54.9%)	
N+	86 (46.0%)	26 (48.1%)	60 (45.1%)	0.682
p16				
Negative	162 (86.6%)	46 (85.2%)	116 (87.2%)	
Positive	25 (13.4%)	8 (14.8%)	17 (12.8%)	0.832
ypT				
T0	160 (85.6%)	47 (87.0%)	113 (85.0%)	
T1	27 (14.4%)	7 (13.0%)	20 (15.0%)	0.678
ypN				
N0	140 (74.9%)	43 (79.6%)	97 (72.9%)	
N+	47 (25.1%)	11 (20.4%)	36 (27.1%)	0.243
PD-L1				
<1	23 (12.3%)	7 (13.0%)	16 (12.0%)	
1-20	96 (51.3%)	27 (50.0%)	69 (51.9%)	
>20	68 (36.4%)	20 (37.0%)	48 (36.1%)	0.947
Radiologic response^				
PR	106 (56.7%)	19 (35.2%)	87 (65.4%)	
CR	81 (43.3%)	35 (64.8%)	46 (34.6%)	0.734

^ PR: partial response; CR: complete response

& p referred to the comparison between ctDNA-guided and traditional groups.

### Treatment

**Figure 2:**
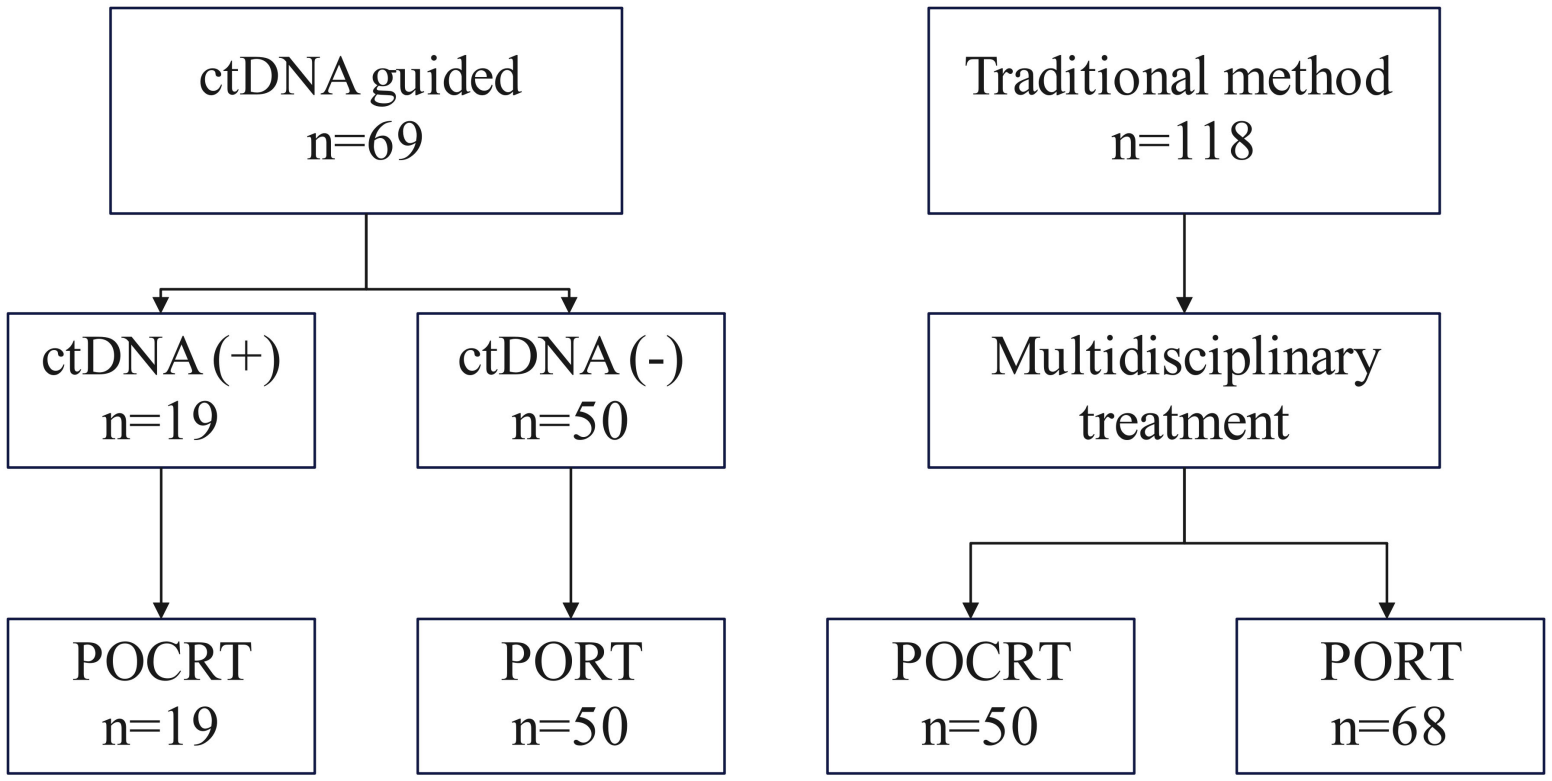
Treatment allocation flowchart. In the ctDNA-guided group, patients received POCRT if post-operative ctDNA was detectable and PORT if ctDNA-negative. In the traditional group, all patients received PORT, with postoperative chemotherapy added per MDT recommendation.

All patients underwent primary tumor resection and neck dissection. Treatment allocation and decision-making differed between the two cohorts. In ctDNA-guided group, 19 (27.5%) patients with detectable post-operative ctDNA received POCRT; while those with undetectable ctDNA (n = 50 of 69) received PORT alone. In traditional group, all patients (n = 118) underwent PORT, with postoperative chemotherapy added based on MDT assessment. The median radiation dose was higher in the ctDNA-guided group (60 Gy; range: 56–66) than in the traditional group (58 Gy; range: 50–66). POCRT utilization was significantly lower in the ctDNA-guided cohort (19/69, 27.5%) compared to the traditional cohort (50/118, 42.4%; p = 0.042) (**Figure 2**).

### Survival analysis

**Figure 3:**
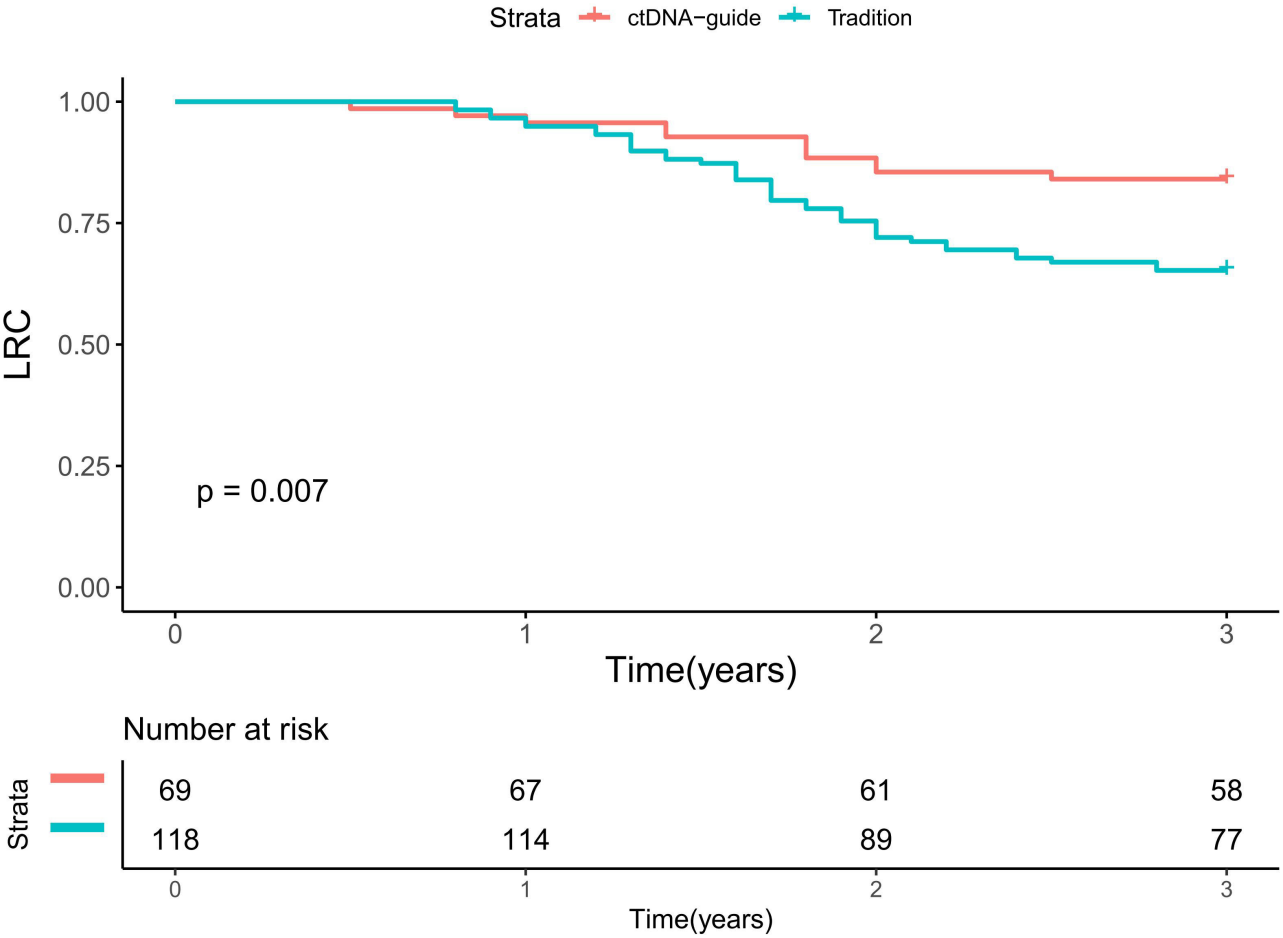
Locoregional control in patients treated by ctDNA-guide approach or traditional procedure.

During a median follow-up period of 2.5 years (range: 0.5-4.5), there were 11 recurrences in the ctDNA-guided group and 41 events in the traditional group. In univariate analyses, the ctDNA-guided procedure demonstrated superior LRC in comparison to the traditional method (**Figure 3**). Significant prognostic predictors included primary site, clinical nodal status, ypN status, and pathological response (all p < 0.05). These factors were subsequently incorporated into a Cox proportional hazards model. In multivariable analysis, the ctDNA-guided program was associated with a 15% (95% CI: 6%-30%) reduction in the risk of locoregional recurrence compared to the traditional procedure. Additionally, patients achieving pCR displayed a 21% (95% CI: 11%-35%) lower risk of locoregional recurrence than those with non-pCR. The ypN+ stage indicated a HR of 1.88 (95% CI: 1.25-3.00), significantly exceeding that of the ypN0 stage (p=0.015). Notably, cN status and primary site did not significantly impact LRC (**Table 2**).

**Table 2:** Univariable and multivariable analysis of predictors for locoregional control.

Variable	Univariable	Multivariable	
		HR [95%CI]	p
Age			
<50			
>=50	0.465		
Sex			
Male			
Female	0.862		
Smoking			
Yes			
No	0.527		
Primary site			
Oral cavity		ref	
Oropharynx		1.87 [0.74-2.66]	0.328
Larynx		1.74 [0.66-2.80]	0.229
Hypopharynx	<0.001	2.31 [0.88-4.36]	0.103
cT			
T3			
T4	0.367		
cN			
N0		ref	
N+	<0.001	2.09 [0.63-5.77]	0.224
p16			
Negative			
Positive	0.332		
ypT			
T0			
T1	0.121		
ypN			
N0		ref	
N+	0.004	1.88 [1.25-3.00]	0.015
PD-L1			
			
1-20			
	0.119		
Radiologic response^			
PR			
CR	0.234		
Pathologic response*			
Non-pCR		ref	
CR	<0.001	0.79 [0.65-0.89]	0.008
Adjuvant therapy			
Tradition		ref	
ctDNA-guide	0.007	0.85 [0.70-0.94]	0.017

^PR, partial response; CR, complete response.

*CR, complete response.

### Subgroup analysis

Patients with detectable ctDNA demonstrated a 4.5-fold higher risk of locoregional failure compared to ctDNA-negative patients (HR 4.50, 95%CI 2.10-12.56, p<0.001). Within the ctDNA-guided group, the administration of POCRT was associated with an HR of 0.73 (95%CI: 0.57-0.83), notably lower than that observed in patients not receiving POCRT (p=0.026). In the traditional group, among patients with positive ctDNA, those receiving POCRT had an HR of 0.87 (95%CI: 0.73-0.93) compared to those not receiving POCRT, with the difference attaining significance (p=0.047). Conversely, in patients exhibiting negative ctDNA, POCRT and no-POCRT groups demonstrated comparable HRs (p=0.193) (**Table 3**).

**Table 3:** Impact of postoperative radiotherapy (PORT) versus postoperative chemoradiotherapy (POCRT) on locoregional control determined by ctDNA presence.

Group	HR [95%CI]	p
Total population (n=187)		
ctDNA negative (n=133)	ref	
ctDNA positive (n=54)	4.50 [2.10-12.56]	<0.001
ctDNA-guide (n=69)		
PORT (n=50)	ref	
POCRT (n=19)	0.73 [0.57-0.83]	0.026
Tradition		
ctDNA positive (n=35)		
PORT (n=19)	ref	
POCRT (n=16)	0.87 [0.73-0.93]	0.047
ctDNA negative (n=83)		
PORT (n=49)	ref	
POCRT (n=34)	0.92 [0.81-1.87]	0.193

### Toxicity

During the neoadjuvant treatment phase, all 187 patients experienced at least one AE, with mucositis (60.9%), vomiting (53.4%), and xerostomia (48.7%) being the most frequent Grade 1/2 toxicities (**Table 4**). Severe (Grade 3/4) AEs occurred less commonly, though mucositis (5.3%), rash (3.2%), and leukopenia (2.1%) were notable exceptions. Immune-related AEs included rash (28.3% overall) and hypothyroidism (20.9%), while potentially serious complications such as pneumonia were rare (1.1%) (**Table 4**).

**Table 4:** Adverse events in the 187 patients during neoadjuvant therapy phase.

Event	Grade 1/2	Grade 3/4
Mucositis	114 (60.9%)	10 (5.3%)
Vomiting	100 (53.4%)	
Xerostomia	91 (48.7%)	
Fatigue	74 (39.6%)	
Rash	53 (28.3%)	6 (3.2%)
Pain	40 (21.4%)	
Hypothyroidism	39 (20.9%)	
Leukopenia	37 (19.8%)	4 (2.1%)
Hypokalemia	25 (13.4%)	
Fever	12 (6.4%)	
Hyponatremia	8 (4.3%)	
Pneumonia	2 (1.1%)	

In adjuvant therapy phase, there were significantly higher toxicity rates in the POCRT group compared to PORT. POCRT patients experienced greater severe xerostomia (10.1% vs 4.2%, p=0.042) and mucositis (21.7% vs 12.7%, p=0.021), with notably higher Grade 3-4 leukopenia (15.9% vs 8.5%, p=0.048). While overall anemia rates were similar between groups (4.3% POCRT vs 2.5% PORT, p=0.372), POCRT showed numerically higher severe events across most categories. Skin toxicity (2.9% vs 0.8%) and thrombocytopenia (2.9% vs 2.5%) differences were not statistically significant (**Table 5**).

**Table 5:** Drug related adverse events between postoperative radiotherapy (PORT) and postoperative chemoradiotherapy (POCRT).

Toxicity	POCRT (n=69)		PORT (n=118)		P*
	Grade 1-2	Grade 3-4	Grade 1-2	Grade 3-4	
Xerostomia	50 (72.4%)	7 (10.1%)	72 (61.0%)	5 (4.2%)	0.042
Mucositis	48 (69.6%)	15 (21.7%)	64 (54.2%)	15 (12.7%)	0.021
Skin	38 (55.1%)	2 (2.9%)	53 (44.9%)	1 (0.8%)	0.185
Anemia	22 (31.9%)	3 (4.3%)	31 (26.3%)	3 (2.5%)	0.372
Leukopenia	20 (29.0%)	11 (15.9%)	37 (31.3%)	10 (8.5%)	0.048
Thrombocytopenia	13 (18.8%)	2 (2.9%)	22 (18.6%)	3 (2.5%)	0.891
Nausea/vomiting	14 (20.3%)	0 (0.0%)	28 (23.7%)	0 (0.0%)	0.593

*Comparison of incidence of each toxicity via the chi-square test.

## Discussion

Our analysis revealed that the ctDNA-guided approach identified a subgroup with superior LRC, it enabled more precise risk-adapted therapy allocation, with ctDNA-positive patients showing particular benefit from POCRT. Notably, drug-related toxicity was less prevalent in the ctDNA-guided program (**Supplementary Table S1**). This study represents the inaugural research illustrating the application of liquid biopsy in patients with HNSCC undergoing neoadjuvant chemoimmunotherapy, potentially facilitating more precise postoperative management for such patients.

The risk of recurrence following cancer treatment had traditionally been evaluated through surgical intervention for solid tumors, relying on a formal histological assessment of the excised specimens. This analysis had served to classify tumor staging and identify any adverse characteristics, which might subsequently necessitated the administration of adjuvant therapy. However, this established protocol had undergone considerable transformation due to the advent of neoadjuvant chemoimmunotherapy. Notably, findings from a groundbreaking single-arm clinical study by Luginbuhl et al. (19) illuminated the potential of neoadjuvant immunotherapy in conjunction with chemotherapy. The incorporation of nivolumab alongside regimens of paclitaxel and carboplatin resulted in a remarkable pCR rate of 49%, with a combined pCR and mPR rate reaching an impressive 65% in patients with locally advanced resectable HNSCC. Furthermore, outcomes from a phase II study investigating the neoadjuvant regimen of treprizumab paired with chemotherapy demonstrated compelling advancements in pathological remission, echoing the substantial effects quantified in previous single-arm studies employing analogous regimens (20). Intriguingly, the neoadjuvant therapy involving immunotherapy in combination with chemotherapy displayed pCR rates of 57.1% and 22.2%, respectively, with corresponding pCR+mPR rates of 92.8% and 22.2%, marking a tantalizing progression from prior findings (21). These results were corroborated in our analysis; however, a new dilemma arose. As data regarding traditional high-risk factors often remain unavailable following postoperative pathology, the standard postoperative management of these patients continues to be uncertain.

ctDNA had emerged as a potentially pivotal indicator for guiding adjuvant therapy and assessing prognosis, garnering considerable attention across various malignancies, albeit with limited focus on HNSCC. Hanna et al. (18) retrospectively evaluated a personalized ctDNA testing methodology, achieving a success rate of 86% across 116 patients. Among these subjects, 75 demonstrated detectable ctDNA prior to treatment; however, no clinical features could reliably predict the detectability or levels of ctDNA. In the cohort of 55 patients assessed post-treatment, 17 were found to be ctDNA positive, revealing that the progression-free survival rate for ctDNA positive patients was significantly inferior to that of their ctDNA negative counterparts, with one-year overall survival rates recorded at 89.1% and 100%, respectively. Honoré et al. (22) further explored ctDNA by employing next-generation sequencing panels encompassing 26 genes and two HPV-16 genes from 53 patients with HNSCC, successfully detecting ctDNA in 41 cases (77%) among pre-treatment samples. Notably, 17 cases (41%) tested positive for ctDNA following treatment. The two-year progression-free survival rate for ctDNA positive patients was 23.5% (ranging from 9.9% to 55.4%), contrasting sharply with the rate of 86.6% (ranging from 73.4% to 100%) for ctDNA negative patients (p<0.05). The median survival time for ctDNA positive patients was 28.4 months (ranging from 14.3 months to an indeterminate duration), whereas the ctDNA negative cohort had not yet reached a median survival time (p=0.011). In another longitudinal study examining ctDNA in 18 HNSCC patients (23), all ctDNA positive individuals experienced relapse post-treatment, while those who were ctDNA negative remained alive and free of recurrence. Flach et al. (24) also documented ctDNA test results from 17 HNSCC patients, noting that postoperative samples revealed detectable ctDNA levels as minimal as 0.0006% of variant allele frequencies. To date, ctDNA had been identified prior to disease progression in all five clinical recurrence cases, with a lead time varying between 108 and 253 days. Importantly, no recurrences were noted in patients with negative ctDNA results. Collectively, these studies signify important preliminary advancements, elucidating the potential utility of ctDNA in HNSCC and advocating for the implementation of targeted multi-gene analysis to inform adjuvant therapy decision-making and recurrence monitoring. Nevertheless, the optimal timing and methodology for ctDNA testing remain poorly delineated. The current study employed a tumor-informed determination method, collecting plasma samples prior to neoadjuvant therapy and 10 to 14 days post-surgery. The results were promising, indicating a 100% detection rate prior to treatment, with only 2% of ctDNA negative samples facing recurrence, contrasted against a 52.6% recurrence rate among ctDNA positive cases. These findings had been corroborated by other studies (25, 26), suggesting that tumor-informed testing possesses superior sensitivity and specificity in ctDNA detection and recurrence prediction compared to alternative methodologies.

MDT approaches had been recognized as a reliable strategy for enhancing prognosis through the adjustment of postoperative therapy programs and schedules, gaining acceptance across numerous medical centers. However, it was observed in the current study that the MDT-based methodology was unexpectedly associated with diminished LRC, highlighting a rather intriguing finding. A potential explanation lied in the fact that traditional MDT evaluations predominantly relied on systemic assessments of high-risk factors, a process that became significantly challenging in cases where a mPR was achieved. Consequently, expert opinions tended to prevail during the deliberations, which may resulted in certain ctDNA positive patients not receiving POCRT. Our findings appeared to resonate with a series of clinical trials concentrating on colon cancer (10–13), which indicated that when compared with the standard methodology, a ctDNA-guided approach led to a reduction in the use of POCRT without compromising recurrence-free survival rates. Furthermore, we observed that the ctDNA-guided strategy decreased POCRT administration by approximately 15%, this variation explained the toxicity difference between the two groups.

POCRT exerted a profound influence on cancer control, with its recommendations derived from two pivotal randomized trials. The RTOG 95-01 trial (27) randomized patients undergoing resection for HNSCC to receive either PORT or POCRT. Preliminary findings after two years of intervention indicated that POCRT significantly improved LRC, although it did not manifest a substantial impact on overall survival. However, a decade later, it became evident that POCRT primarily benefited patients exhibiting extranodal extension or tangential margins. Conversely, the EORTC 22931 trial (28) encompassed a broader spectrum of high-risk patients who were randomized to receive either POCRT or PORT. This distinctive study revealed that after five years of POCRT treatment, patients experienced no progression, and overall survival rates improved. The researchers elucidated that POCRT predominantly enhanced disease-free survival and overall survival compared to patients with three or more positive lymph nodes, rather than affecting the rates of lymph node involvement. In patients classified as stage IV, the incorporation of chemotherapy into radiotherapy significantly lowered the risk of recurrence by 24% and decreased the mortality rate by 20%. While these studies exhibited considerable reliability, their conclusions may not extend to patients with mPR or pCR HNSCC lacking adverse pathological characteristics. To our knowledge, our study represents the first exploration of POCRT in mPR HNSCC patients. Among the 35 ctDNA-positive patients in traditional therapy cohort, the addition of chemotherapy to PORT appeared to enhance disease control (13% absolute risk reduction), while no similar benefit was observed in ctDNA-negative patients (n=83). These findings should be interpreted cautiously given the modest sample size of the ctDNA-positive subgroup. This revelation is of great significance, as it underscores the recognition of a novel indicator for POCRT implementation.

Limitation in current study must be acknowledged, first, this study lacked randomization, it might affected our analysis; second, owing to the limited follow-up time, we could not obtain enough disease specific or overall survival; third, our sample size was relatively small, more studies were required; fourth, inconsistent follow-up intervals (ranging from 6-12 months) might affect the exact time of locoregional failure; fifth, the absence of standardized MDT criteria for POCRT administration represented significant methodological constraints.

In summary, ctDNA status effectively stratified recurrence risk in HNSCC patients achieving mPR following neoadjuvant chemoimmunotherapy, with detectable post-treatment ctDNA identifying a high-risk subgroup that showed improved locoregional control when treated with POCRT compared to PORT. While these results suggest ctDNA may guide adjuvant therapy intensification, the non-randomized design and modest sample size warrant validation in prospective trials.

## Data Availability

The original contributions presented in the study are included in the article/**Supplementary Material**. Further inquiries can be directed to the corresponding author/s.
