# Correction: Evaluation of ctDNA-guided adjuvant therapy de-escalation in head and neck squamous cell carcinoma: a comparative cohort study

**DOI:** 10.3389/fimmu.2026.1845587

**Published:** 2026-05-08

**Authors:** Xu Zhang, Qigen Fang, Junhui Yuan, Liyuan Dai, Ruihua Luo, Tao Huang, Yinfei Wu

**Affiliations:** 1 Department of Head Neck and Thyroid, The Affiliated Cancer Hospital of Zhengzhou University & Henan Cancer Hospital, Zhengzhou, China; 2 Department of Radiology, The Affiliated Cancer Hospital of Zhengzhou University & Henan Cancer Hospital, Zhengzhou, China; 3 Department of Breast, The Affiliated Cancer Hospital of Zhengzhou University & Henan Cancer Hospital, Zhengzhou, China; 4 Moores Cancer Center, University of California, San Diego, San Diego, CA, United States; 5 Health Management Center, The Affiliated Cancer Hospital of Zhengzhou University & Henan Cancer Hospital, Zhengzhou, China

**Keywords:** head and neck squamous cell carcinoma, CtDNA, neoadjuvant chemoimmunotherapy, adjuvant chemotherapy, pathologic complete response

In the original publication, the methodology for the personalized, tumor-informed *ctDNA testing* was incorrectly stated as being performed “using the Signatera™ (Natera, Inc.) assay.

A correction has been made to the section (*ctDNA testing* paragraph in **Methods** section, Page 3):

“This methodology aligns with previously published protocols (18). ctDNA was detected in plasma samples gathered prior to neoadjuvant chemoimmunotherapy and within 10–14 days postsurgery. Personalized, tumor-informed ctDNA testing was performed using Illumina (NextSeq 500/NovaSeq 6000) next-generation sequencing platform (Wanlei, Shenyang). Testing was conducted by our core facility, utilizing matched tumor tissue and blood samples to design patientspecific assays. Somatic mutations were identified via multiplex PCR amplification via a 16-plex PCR panel targeting tumor-derived variants, enabling bespoke detection with a sensitivity of 0.01% variant allele frequency. The bioinformatics pipeline included alignment (BWA-MEM), duplex consensus sequencing to reduce noise, and variant calling via a proprietary algorithm, with germline filtering using matched white blood cell-derived DNA. A plasma sample was classified as ctDNA-positive if ≥1 somatic variant was detected. Variant persistence in postoperative samples was confirmed if: the variant was detected pre-treatment, present in the binary comparison map file, or observed in ≥3 reads in postoperative plasma. ctDNA level was recorded with units of mean tumor molecules (MTM) per mL of plasma.”

The original article has been updated.

