# Supplementary material for: Evaluation of ctDNA-guided adjuvant therapy de-escalation in head and neck squamous cell carcinoma: a comparative cohort study
Source: Front Immunol. 2025 Aug 7;16:1576042. doi: 10.3389/fimmu.2025.1576042 (PMC12367698; doi:10.3389/fimmu.2025.1576042)
Supplement: Supplementary file 1 [file Table1.doc]

Supplementary Table 1. Drug related adverse events between the two groups.

| Toxicity | ctDNA-guide (n=69) | | Tradition (n=118) | | P* |
| --- | --- | --- | --- | --- | --- |
|  | Grade 1-2 | Grade 3-4 | Grade 1-2 | Grade 3-4 |  |
| Xerostomia | 45 | 4 | 77 | 8 | 0.881 |
| Mucositis | 40 | 8 | 72 | 22 | 0.119 |
| Skin | 28 | 1 | 63 | 2 | 0.085 |
| Anemia | 18 | 2 | 35 | 6 | 0.542 |
| Leukopenia | 15 | 3 | 42 | 18 | 0.008 |
| Thrombocytopenia | 10 | 0 | 25 | 5 | 0.103 |
| Nausea/vomiting | 10 | 0 | 32 | 0 | 0.046 |

* Comparison of incidence of each toxicity via the chi-square test.
